# The long-term health effects of attending a selective school: a natural experiment

**DOI:** 10.1186/s12916-020-01536-7

**Published:** 2020-04-03

**Authors:** Jessica Butler, Corri Black, Peter Craig, Chris Dibben, Ruth Dundas, Michelle Hilton Boon, Marjorie Johnston, Frank Popham

**Affiliations:** 1 Centre for Health Data Science, University of Aberdeen, Aberdeen, AB25 2ZD UK; 2 MRC/CSO Social and Public Health Sciences Unit, University of Glasgow, 200 Renfield Street, Glasgow, G2 3AX UK; 3 Institute of Geography, University of Edinburgh, Drummond Street, Edinburgh, EH8 9XP UK

## Abstract

**Background:**

Education is widely associated with better physical and mental health, but isolating its causal effect is difficult because education is linked with many socioeconomic advantages. One way to isolate education’s effect is to consider environments where similar students are assigned to different educational experiences based on objective criteria. Here we measure the health effects of assignment to selective schooling based on test score, a widely debated educational policy.

**Methods:**

In 1960s Britain, children were assigned to secondary schools via a test taken at age 11. We used regression discontinuity analysis to measure health differences in 5039 people who were separated into selective and non-selective schools this way. We measured selective schooling’s effect on six outcomes: mid-life self-reports of health, mental health, and life limitation due to health, as well as chronic disease burden derived from hospital records in mid-life and later life, and the likelihood of dying prematurely. The analysis plan was accepted as a registered report while we were blind to the health outcome data.

**Results:**

Effect estimates for selective schooling were as follows: self-reported health, 0.1 worse on a 4-point scale (95%CI − 0.2 to 0); mental health, 0.2 worse on a 16-point scale (− 0.5 to 0.1); likelihood of life limitation due to health, 5 percentage points higher (− 1 to 10); mid-life chronic disease diagnoses, 3 fewer/100 people (− 9 to + 4); late-life chronic disease diagnoses, 9 more/100 people (− 3 to + 20); and risk of dying before age 60, no difference (− 2 to 3 percentage points). Extensive sensitivity analyses gave estimates consistent with these results. In summary, effects ranged from 0.10–0.15 standard deviations worse for self-reported health, and from 0.02 standard deviations better to 0.07 worse for records-derived health. However, they were too imprecise to allow the conclusion that selective schooling was detrimental.

**Conclusions:**

We found that people who attended selective secondary school had more advantaged economic backgrounds, higher IQs, higher likelihood of getting a university degree, and better health. However, we did not find that selective schooling *itself* improved health. This lack of a positive influence of selective secondary schooling on health was consistent despite varying a wide range of model assumptions.

## Background

Education is associated with physical and mental health and life expectancy in countries around the world, and the role of education in improving health is a focus of diverse research and global policy [1–6]. However, there is vigorous debate about the causal effects of educational reforms. Because children from socio-economically advantaged families are more likely to attend better-resourced schools, be educated longer, and gain more qualificatio ns, the association between education and health may simply reflect background advantages rather than effects of educational policy itself [4, 7–9].

Studies seeking to identify the true causal effects of education on health have focused on evaluating increases in length of schooling. They take advantage of policy decisions that create “natural experiments”, or environments that mimic the conditions of a randomised trial [10]. One such natural experiment is a nationwide increase in the age at which a child may legally leave school, which allows the comparison of the health of the cohorts just before and just after the policy was implemented. Analyses of these natural experiments across multiple countries have shown that length of compulsory education causes small but significant reductions in mortality, smoking, and obesity [11–14].

The long-term effects of other educational reforms have received less attention. Here we consider the policy of stratifying students based on their academic ability, referred to as selective schooling. Historically, most developed nations stratified students by ability either into separate schools or classes, but since the 1960s many countries moved to less stratification [15, 16]. Recently, however, the policy has come back into debate, and across OECD nations, there have been reforms both towards a more stratified system and away from it [17]. In the UK, the educational system moved from selective to comprehensive more than 40 years ago [18, 19], but the present UK government has pledged a return to a stratified system in an effort to reduce the achievement gap between children of different socioeconomic circumstances [20].

Previous analyses of selective schooling’s effects have focused on its impact on educational trajectories and long-term economic outcomes [21–24]. In contrast, selective schooling’s effects on health have been less well explored. The mechanisms by which selective schooling may change health are proposed to be both direct, via health literacy, as well as indirect via changes in prosperity, status, and control [25–27]. However, while studies have demonstrated an association between selective schooling and health, the results have been shown to be subject to confounding. Recent work has attempted to model the association more robustly by controlling for a wide variety of covariates and matching exposed and unexposed individuals [28–30]. However, in addition to the potential of residual confounding, these studies evaluated only self-reported health measures derived from participant survey. The results are hampered both by surveys only through mid-life and by high attrition over time leaving a non-representative population.

Here we take advantage of a natural experiment in school assignment that occurred in Britain in the 1960s, as well as the data sharing policy of the National Health Service of Scotland to evaluate the long-term causal effects of selective schooling on health. Our study population is 8087 people who were assigned to selective or non-selective secondary school based on their performance on an exam taken at age 11. They are part of the Aberdeen Children of the 1950s, a long-running birth cohort which holds extensive data on participants from across their lives [31, 32]. As this population was one of the last to be assigned to school via exam, and as they are now over 60, they provide an excellent opportunity to evaluate long-term health effects. We show that a regression discontinuity design which alleviates the bias due to residual confounding is appropriate for estimating the effect on health of attending a selective secondary school. We consider a wider range of outcomes than previous studies, including self-reported physical health and mental health, diagnoses of 30 chronic conditions from hospital records, and premature death. We consider health at two periods: when participants were in middle age and early old age.

## Methods

### Study design

A detailed study design was pre-registered and accepted by BMC Medicine as a registered report after peer review [33]. We were blind to participant outcomes before study acceptance.

This analysis uses a regression discontinuity design to investigate the effect of selective secondary schooling on health [34, 35]. In such a design, allocation to an exposure depends on the value of a continuous variable (the forcing variable). In this study, assignment to selective schooling depended on an exam score at age 11.

The design assumes that if there were no treatment effect, there would be a smooth relationship between the forcing variable and the outcome. In this study, if selective secondary schooling has no effect on health, the association of health outcomes will be smooth across exam scores. However, if there is a discontinuity in this smooth relationship at the forcing variable cut-point (i.e. if health outcomes change suddenly in people with scores close to the cut-point), the effect can be attributed to selective schooling. We designed the analysis based on recommendations in Cattaneo, Idrobo and Titiunik [36].

### Data sources

We used data previously collected by the Aberdeen Children of the 1950s birth cohort study linked to NHS Scotland routinely collected hospital admissions data.

### Exposure

In the UK in the 1960s, there were two types of state-run secondary schools: junior secondary (called secondary modern schools in England) designed to prepare students for training and work, and senior secondary (grammar schools in England) designed to prepare students for further education at university and professional careers [37].

Students were allocated to secondary school based on their performance on a series of tests taken in the final year of primary school, referred to as the 11+ exam [19]. For Aberdeen Children of the 1950s participants, those with 11+ exam scores below 540 were assigned to junior secondary schools; those with scores at or above 560 were assigned to senior secondary schools; and those in between were considered individually [38].

### Outcomes

We considered the following six measures as outcomes:

1. Overall health score (self-reported in 2001)
2. Mental health score (self-reported in 2001)
3. Presence of limitation due to health problems (self-reported in 2001)
4. Number of chronic diseases (from hospital records 1997–2001)
5. Number of chronic diseases (from hospital records 2011–2015)
6. Risk of death by age 60 (from national death records)

The first three outcomes were collected by postal survey of the cohort in 2001 when they were between 46 and 51 years old [32].

The two chronic disease burden outcomes were derived from hospital and mental health admission records (NHS Scotland Information Service Division datasets SMR01 and SMR04 [39]). Diagnoses of any of the chronic conditions validated by Tonelli [40] were counted. Two 5-year windows were analysed, the first corresponding to the participants’ mid-life (46–51 years old) at the time as when they were surveyed on their health, and the second corresponding to early old age (60–65 years old).

Date of death is held by the Aberdeen Children of the 1950s study and is updated quarterly from the death certificates at National Records of Scotland [32].

In addition to the above health outcomes, we also report analysis of the known positive control outcome of probability of completing a higher degree [23].

### Population exclusions

There are 12,148 people in total in the Aberdeen Children of the 1950s study—the population is all the children born in Aberdeen between 1950 and 1955 who attended primary school there. The 11+ exam and assignment procedure were changed the year the youngest students in the Aberdeen Children of the 1950s cohort took it, so we excluded these students. We also excluded students who were already enrolled in primary schools that were selective, religious, for special needs, or for children in care, as these students were highly likely to stay in the same type of secondary school regardless of their 11+ scores. Finally, we excluded students who did not take all five parts of the 11+ exam. These exclusions were previously declared in the registered report. The remaining population is our base sample (*n* = 8087), comprising all Aberdeen Children of the 1950s cohort members assigned to secondary school based on the same 11+ test score criteria (Table 1).

**Table 1:** Exclusions from the total Aberdeen Children of the 1950s population (*n* = 12,148) for this study

In youngest school year (different exam)	2513
In private primary	216
In selective state-run primary	531
In Roman Catholic primary	260
In special needs primary	183
In special care outside Aberdeen	76
Lack 11+ scores	282
**Total base sample**	**8087**
Lack secondary school attendance	3048
**Total study sample**	**5039**

**Fig. 1:**
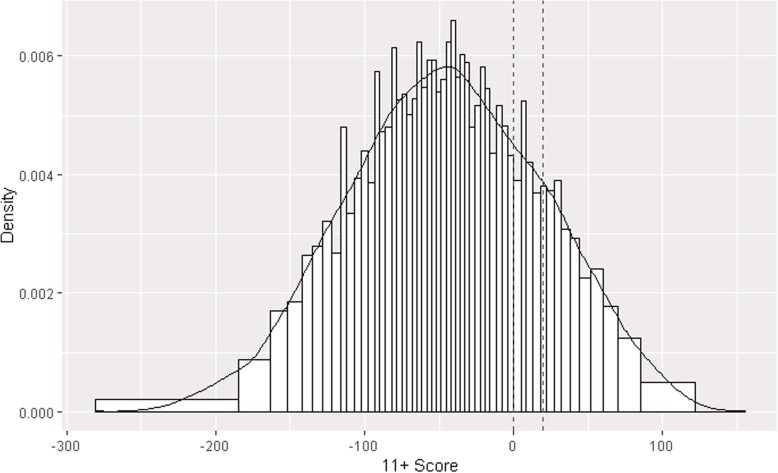
Distribution of 11+ exam scores. Population is the study sample (*n* = 5039) divided into 100-person quantiles. Dashed lines show the primary and secondary cut-points of 540 and 560 (rescaled to 0 and 20)

**Fig. 2:**
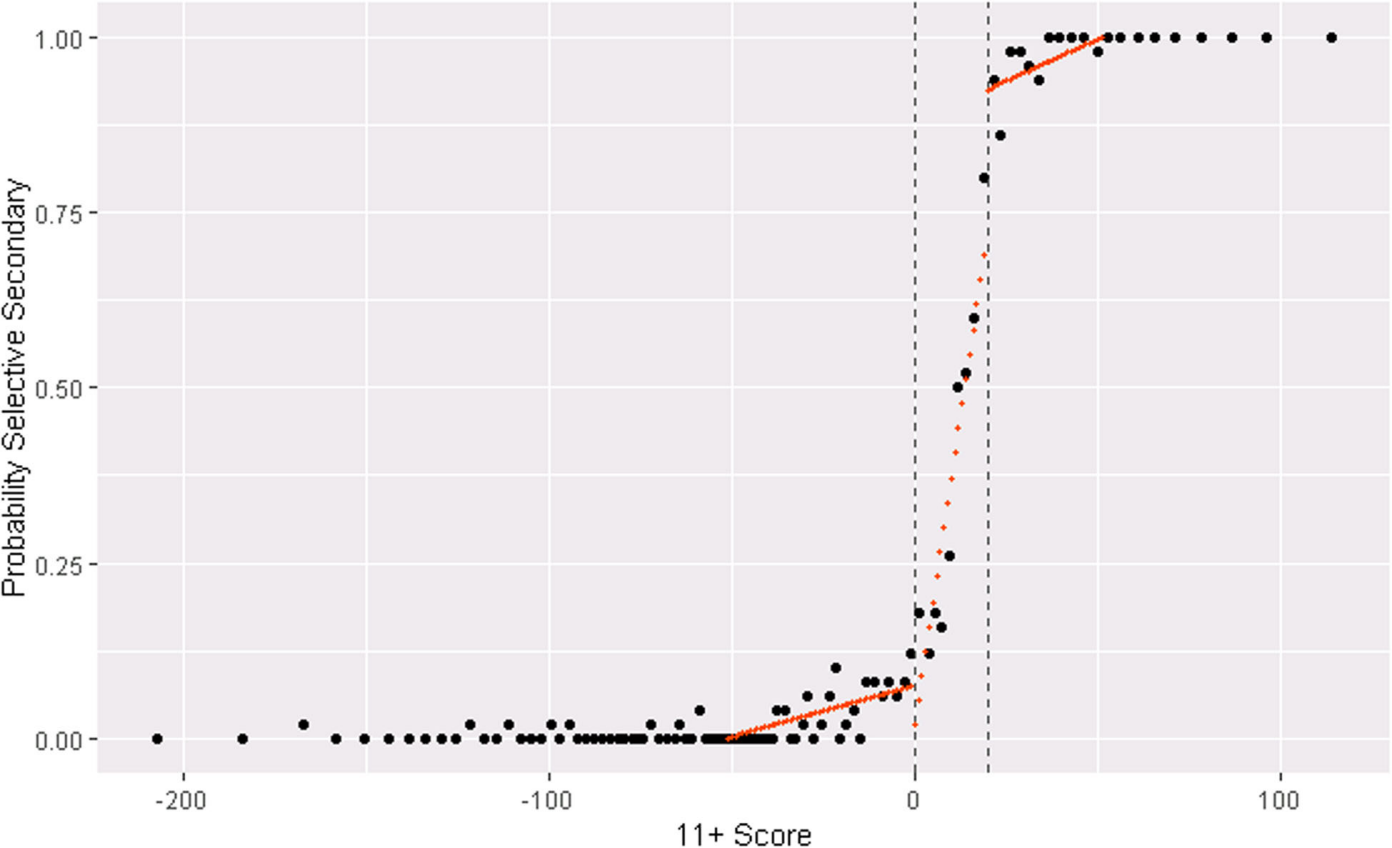
Probability of attending a selective secondary school. Population is the study sample (*n* = 5039) with points showing mean scores for groups of 50 people. Dashed lines show the primary and secondary cut-points of 540 and 560 (rescaled to 0 and 20). Red lines are predicted probabilities of attending a selective secondary school within the population bandwidth of 76 points to either side of the primary score cut-point

In the registered report, we stated that type of secondary school attended was known for 5112 of those in the base sample. This was incorrect. We had incorrectly included 73 people for whom secondary school is recorded as “outside Aberdeen” in the non-selective secondary school population, but for these people, there is no information about their schools’ selectivity. Here we have excluded them. Figures 1 and 2 in the registered report are re-created here with the corrected study population, and exclusion of these 73 did not alter the results presented below. After this correction, there were 5039 individuals in the base sample for whom secondary school type is known. This is our study sample. Descriptive statistics comparing the total Aberdeen Children of the 1950s population base sample and the study sample are presented in Table 2.

**Table 2:** Descriptive statistics

	Aberdeen Children of the 1950s (total)		Base sample (total)		Study sample (total)		Study sample (attended non-selective secondary)		Study sample (attended selective secondary)	
	Cases	Mean	Cases	Mean	Cases	Mean	Cases	Mean	Cases	Mean
Boy (%)	12,148	52%	8087	52%	5039	49%	3845	49%	1194	49%
IQ aged 9	11,384	111	7998	110	4998	112	3816	106	1182	129
Father non-manual labour (%)	12,148	21%	8087	17%	5039	17%	3845	13%	1194	32%
11+ exam score	9600	498	8087	495	5039	502	3845	476	1194	584
General health (0–3 scale)	7143	2.0	4806	2.0	4734	2.0	3542	1.9	1192	2.2
Mental health (0–15 scale)	7111	8.5	4786	8.5	4716	8.5	3529	8.5	1187	8.7
Life limited by health (%)	7012	17%	4720	17%	4651	17%	3470	18%	1181	13%
Linked to NHS records (%)	10,634	88%	7181	89%	4687	93%	3614	94%	1073	90%
Chronic diagnoses 1997–2001 (per 100 people)	10,634	13	7181	13	4687	11	3614	13	1073	8
Chronic diagnoses 2011–2015 (per 100 people)	10,634	39	7181	41	4687	40	3614	44	1073	26
Died before age 60 (%)	12,148	8%	8087	8%	5039	4%	3845	5%	1194	3%
Attended selective secondary (%)	na	na	na	na	5039	24%	na	na	na	na

### Missing data

Lack of survey response and NHS dataset limitations mean all outcomes are not available for all participants, so we consider the results of 6 outcomes from 3 sources. Probability of responding to the 2001 survey increased with increasing 11+ exam scores. We impute school attendance for the 3048 people in the base sample for whom secondary attendance is not known using their 11+ exam scores, and present the effect estimates for the entire base population as a sensitivity analysis below. Probability of being linked to NHS records was higher and more stable and decreased slightly with increasing test scores. Death records include those who died in the UK and abroad. Complete cases for all outcomes are shown in Table 2.

### Variable processing

Details of the variables received from the Aberdeen Children of the 1950s study or from NHS hospital admissions records are given in Additional Table 1.

#### Schools and scores

Primary school attended is defined as the school attended in 1962 (variable RS028). Secondary school attended is available from the postal survey in 2001 (variable Q1094), with the oldest students in the cohort also having had it recorded from their education records in 1964 (variable RS095). There were no discrepancies between sources, so both were used. Selective schools were defined as the three state-run schools which required a high 11+ exam score to attend and the three fee-paying schools which also required a high admission test score to attend. The 11+ exam score is the sum of variables RS053-RS057, rescaled so the 540 cut-point is 0.

#### Self-rated health

Overall health score is from a self-rated 4-point scale (variable Q1001), and limitations of daily activities due to health is from a self-rated presence/absence (variable Q1002). Mental health is the sum of four survey questions each on a 4-point scale, so that mental health score ranges from 0 to 15. For both general health and mental health, a higher score is better health.

#### Chronic disease diagnoses

For this study, processing of hospital and mental health admission records was done by the Information Services Division (ISD) of NHS Scotland, as data protection laws require. ISD linked Aberdeen Children of the 1950s study participants via their Community Health Index number to the national hospital and mental health admission datasets SMR01 and SMR04. Each admission record includes diagnoses coded according to the International Statistical Classification of Diseases (ICD) [41]. We supplied ISD with Tonelli’s set of chronic diseases and their validated ICD diagnosis codes [40] (Additional Table 1). For each individual, ISD returned a binary variable for each disease indicating presence/absence of any of the ICD codes in that disease’s set within the given 5-year window (1997–2001 or 2011–2015). We summed the number of chronic diseases diagnosed during each window to one variable. ISD also provided a variable indicating if the participant had no Community Health Index number and therefore could not be linked to medical records.

#### Death

Death records are available for all participants in the UK and those who were reported from abroad for all years since study inception. Age of death was derived from the date of death and the age in months in December 1962.

#### Covariates

Father’s occupation at child’s birth has previously been classified using The Registrar General’s Classification of Occupations 1950 [38]. Father’s occupation (variable RS040) was considered a non-manual labour if his occupation was coded class I or II. IQ at age 9 was given by variable RS052.

### Hypotheses

The pre-registered hypotheses were as follows:

- (H0) Selective secondary school attendance is not associated with self-reported overall health in mid-life.
- (H1) Selective secondary school attendance is not associated with self-reported mental health in mid-life.
- (H2) Selective secondary school attendance is not associated with limiting health problems in mid-life.
- (H3) Selective secondary school attendance is not associated with chronic disease diagnoses in mid-life.
- (H4) Selective secondary school attendance is not associated with chronic disease diagnoses in later life.
- (H5) Selective secondary school attendance is not associated with risk of death by age 60.

### Primary model

As registered, our primary modelling strategy modelled the schooling-health effect using a non-parametric, linear approximation local to the score cut-point. This method considers a bandwidth of scores around the cut-point and fits a regression function for each population on either side of the cut-point, giving more weight to observations closer to the cut-point. Bandwidth was chosen automatically using the mean square error optimal method [42] and a triangular kernel function used to assign weights to the observations within the bandwidth. The difference in outcome due to selective schooling is then the difference of the intercepts at the cut-point.

To account for the fuzziness of the score-assignment relationship, we estimated the effect of selective schooling as the ratio of the effect given the probability of attendance (equivalent to local average treatment effect estimation in randomised controlled trials) [35, 36]. To do so, we used a two-stage least squares method, where the first stage was a model of the probability of attending a selective school contingent on exam score, the cut-points and their interaction. This probability of treatment was then used instead of a binary treatment variable in the second stage.

### Sensitivity analyses

The registered secondary analyses for each outcome were to report effect estimates for (1) variation in bandwidth selection, (2) higher-order polynomial form, (3) inclusion of covariates (sex, IQ score at age 9, father’s occupation), (4) exclusion of the population immediately around the cut-off, and (5) inclusion of those for whom secondary school attended is not known (imputed by inverse-probability weighting using the 11+ score).

### Power

As detailed in the registered report, analysis of statistical power for regression discontinuity designs suggests that to detect a small difference (0.2 of a standard deviation) between two means at the 2.5% level (one-tailed) with 95% power would require a sample size of 1753, with 80% power for a sample size of 1078. For a large effect size (0.8 of a standard deviation), the sample sizes were 109 and 68 respectively. These results suggest that regression discontinuity designs’ sample size is around 2.7 times higher than the corresponding RCT [43].

Average self-rated health in the Aberdeen Children of the 1950s study was 2 with a standard deviation of 0.8. For a small effect (0.2 of a standard deviation) at the 5% level with 80% power and taking account of the relative size of the exposure groups, the regression discontinuity sample size applying the correction is 2762, and for a large effect (0.8 of a standard deviation), it is 201.

Given the assumptions that underlie these estimates, for this design it is prudent to assess the results in totality rather than focussing on the achievement of statistical significance at a given level on any one test. We assess whether effect sizes were consistent across outcomes and robust across sensitivity analyses, whether the magnitude of the effect sizes is plausible, and whether the conclusions are in line with other studies.

### Software

Two-stage least squares was done in R using the package AER [44] and bandwidth optimisation and McCrary tests done with the package rddapp [45]. The metadata and all code used for analysis and generation of figures and tables are included (Additional file 2).

## Results

### Validity of the design

The study design assumes that in the absence of an effect of selective schooling, there would be a smooth relationship between the 11+ exam score and health. In contrast, the presence of an effect of selective schooling would be seen as a discontinuity at the score cut-point—there would be a change in the mean level of health between people who scored just to either side of the cut-point.

To determine if the study represents a natural experiment that can be analysed for a regression discontinuity, we conducted several tests of validity. These tests are all based around the idea that aside from school assignment, there should be no other discontinuities of study population characteristics across the cut-point, including in the covariates or in attrition over time.

The distribution of the 11+ assignment scores in the study sample was smooth across both cut-points (McCrary density function test of discontinuity [46] at score of 0, *p* = 0.76 at score = 20, *p* = 0.50) indicating no manipulation of the scores to influence school assignment for children close to the cut-point (Fig. 1).

For a valid design, confounders (sex, IQ at age 9, father’s occupation at birth) should also be smooth across the assignment cut-point. Discontinuities in these variables would call into question the validity of the test score as a forcing variable for school attendance [36]. IQ at age 9, probability of being a boy, and probability of father’s occupation being non-manual are all smooth across the range of 11+ exam scores (Additional Figure 1). In addition, measuring the discontinuity of these confounders at the cut-point using the primary model did not find evidence for any difference (Table 3 and Additional Figure 1). Altering the bandwidth used in the model did not change these results although the smallest bandwidth estimates are non-zero but very imprecise (Additional Figure 1).

**Table 3:** Estimated effect of attending a selective secondary school on confounding variables

	Effect size	95% CI	*p* value	*n*	Bandwidth
IQ age 9	−0.14	(− 1.49 to 1.21)	0.84	3441	77
Boy	−0.01	(− 0.08 to 0.06)	0.82	4330	111
Father non-manual occupation	0.02	(− 0.05 to 0.09)	0.63	3570	79
Linked to NHS records	0.00	(− 0.03 to 0.03)	0.93	4892	163

Similarly, probability of replying to the 2001 postal survey or being linked to NHS records (the sources of the outcome data) should be smooth across the assignment cut-point. Likelihood of replying to the survey increased for increasing 11+ score but was smooth across the cut-point [33]. Probability of being linked to NHS records decreased slightly with increasing 11+ score but selective secondary school attendance did not affect it (Table 3). We analysed potential confounding and attrition bias by controlling outcome models for confounding and by weighting using inverse-probability weighting for the likelihood of replying to the survey (results below).

The 11+ score was a strong but not perfect predictor of selective school assignment (Fig. 2). In the registered report, we declared we would use a single cut-point when modelling this first stage, but this gave fairly poor estimates of probability of attending secondary school because the slope of relationship between 11+ score and secondary school attendance changed at both points. For the analysis here, we use both cut-points to estimate the first stage probability of attending a selective school (Fig. 2). The second stage estimates remain local treatment effects for an 11+ score at the primary cut-point of 0.

### Effect of selective schooling on self-reported health in mid-life

Study members reported on three measures of their health by survey when they were in their late 40s to early 50s—their general health, their mental health, and whether they had an illness that limited their lives.

**Fig. 3:**
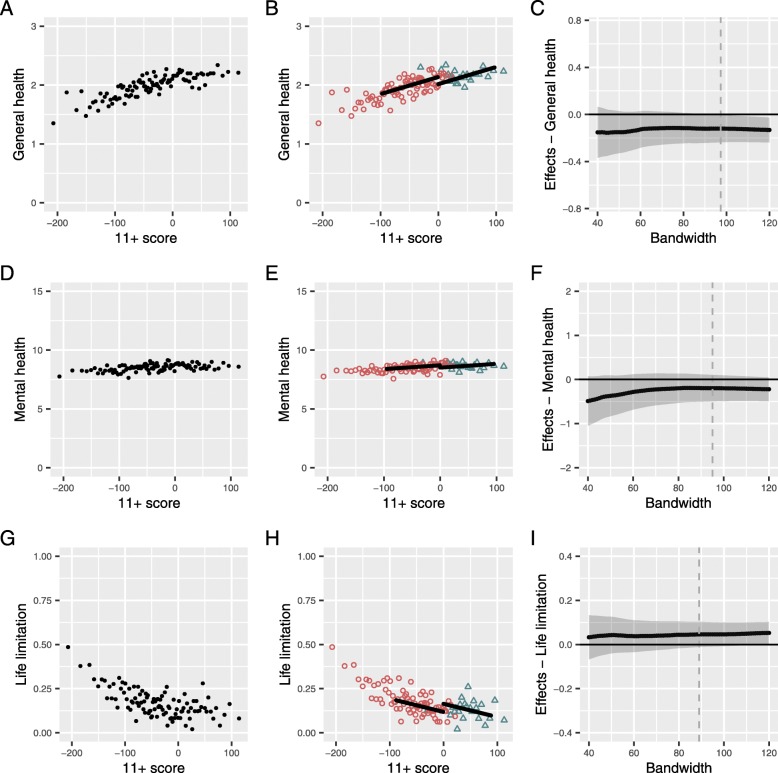
Assessment of the effect of selective schooling on the self-reported health outcomes. Population is the study sample (*n* = 5039) and points are means of 50 people. **a**, **d**, **g** The first column shows the 11+ exam score vs the outcome. **b**, **e**, **h** The second column shows the same data with the population separated by selective (open orange triangles) and non-selective (open blue circles) secondary school attendance. The line shows the estimated effect size of secondary school attendance at the cut-point extrapolated across the optimal bandwidth. **c**, **f**, **i** The third column shows re-estimates of the effect size of secondary school attendance for a range of population sizes around the cut-point (bandwidth sizes). The effect size estimate at the predicted optimal bandwidth (reported in Table 4) is shown as a dashed line

For all three outcomes, health was better as 11+ scores increased (Fig. 3a, d, and g) and those who attended selective school had better mean scores (Table 2).

However, isolating the effect of selective schooling itself (modelled as linear within the predicted optimal bandwidth and weighting the responses of those closer to the cut-off more highly) showed that people who attended selective schools reported slightly poorer general health than we would expect given their test score (Fig. 3b). The effect of selective schooling was − 0.12 on the 4-point scale of general health (0.15 of a standard deviation poorer) (Table 4).

**Table 4:** Estimated effect of attending a selective secondary school on health outcomes

	Effect size	95% CI	*p* value	*n*	bandwidth
General health (0–3 scale)	−0.12	(− 0.24 to 0.00)	0.04	3891	97
Mental health (0–15 scale)	−0.20	(− 0.50 to 0.10)	0.20	3824	95
Life limited by health	0.05	(− 0.01 to 0.10)	0.12	3632	89
Chronic disease diagnoses per person 1997–2001	−0.03	(− 0.09 to 0.04)	0.46	3238	78
Chronic disease diagnoses per person 2011–2015	0.09	(− 0.03 to 0.20)	0.13	4138	119
Died before age 60	0.00	(− 0.02 to 0.03)	0.74	4821	151

The effect of selective schooling on mental health and life limitation were also negative; however, these estimates were imprecise with confidence interval including positive effects as well (Table 4 and Fig. 3e, h). The effect estimate for mental health was − 0.2 on the 16-point scale (0.10 of a standard deviation poorer). The estimate for probability of having a life limitation was 5 percentage points higher (0.14 of a standard deviation more likely).

### Effect of selective schooling on chronic disease burden in mid-life

We also assessed health at mid-life using hospital admission records. We calculated the number of unique chronic diseases diagnosed in all hospital admissions during the 5 years before participants reported their health values given above.

**Fig. 4:**
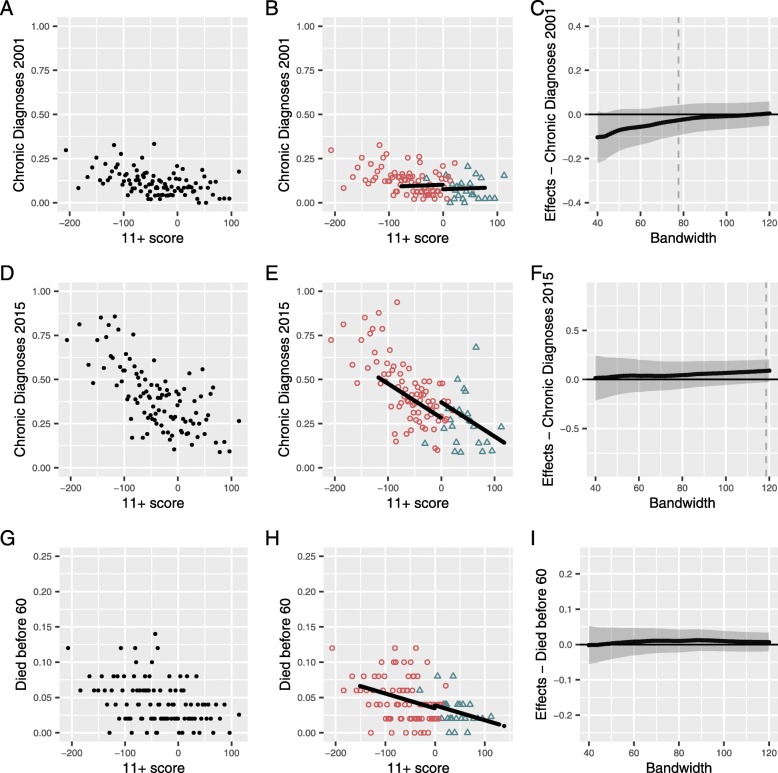
Assessment of the effect of selective schooling on outcomes derived from hospital and death records. Population is the study sample (*n* = 5039) and points are means of 50 people. **a**, **d**, **g** The first column shows the 11+ exam score vs the outcome. **b**, **e**, **h** The second column shows the same data with the population separated by selective (open orange triangles) and non-selective (open blue circles) secondary school attendance. The line shows the estimated effect size of secondary school attendance at the cut-point extrapolated across the optimal bandwidth. **c**, **f**, **i** The third column shows re-estimates of the effect size of secondary school attendance for a range of population sizes around the cut-point (bandwidth sizes). The effect size estimate at the predicted optimal bandwidth (reported in Table 4) is shown as a dashed line

As with the self-reported health outcomes, chronic disease burden in mid-life was better (lower) with increasing 11+ scores (Fig. 4a) and those who attended selective schools had a lower mean number of diagnoses (Table 2). The estimated effect of selective schooling itself on the number of chronic disease diagnoses at mid-life was 3 fewer diagnoses per 100 people (0.07 of a standard deviation) but imprecise with confidence interval including more diagnoses as well (95% CI 9 fewer to 4 more per 100 people) (Table 4 and Fig. 4b).

### Effect of selective schooling on chronic disease burden in later life and on premature death

To evaluate health in later life, we also calculated the number of unique chronic diseases diagnosed across all hospital admissions from 2011 to 2015 when the participants were in their early to mid-60s.

The chronic disease burden had more than tripled across the population (Table 2), and the number of diagnoses decreased steeply with increasing 11+ scores compared to mid-life (Fig. 4d). The mean burden remained lower in those who attended selective schooling (Table 2).

However, the effect of selective schooling itself on number of chronic disease diagnoses in later life was estimated to be an additional 9 diagnoses per 100 people (0.10 of a standard deviation), though the estimate was imprecise with confidence interval including fewer diagnoses as well (95% CI between 3 fewer and 20 more diagnoses per 100 people) (Fig. 4e and Table 4).

We also evaluated the likelihood of dying before age 60. Overall, there was a low chance of premature death (4%) that decreased with increasing 11+ score, with the mean likelihood halved in those attending selective schooling (Fig. 4g and Table 2).

The estimated effect of selective secondary school attendance on premature death was no difference though the estimate was imprecise (95% CI from 2 percentage points lower to 3 percentage points higher) (Table 4 and Fig. 4h).

### Effect of selective schooling on completing a higher degree

To test our primary model against a known positive control, we evaluated the effect of selective schooling on the likelihood of attaining a higher degree. At the cut-point, attending a selective secondary school increased the probability of attaining a higher degree by 16 percentage points (95% CI 10 to 22 percentage points).

### Sensitivity analyses

Five parameters were varied to test the robustness of the estimated effects of secondary schooling. These were as follows: (1) the population size used to make the model, (2) the weight given to individuals nearer the cut-point, (3) the use of second or third order polynomials of the 11+ score in the model, (4) the inclusion of confounders (sex, IQ at age 9 and father’s occupation at birth), and (5) the inclusion by imputation of the population whose secondary school attendance was not known.

For each of the outcomes presented above, the population around the cut-point used to construct the model (referred to as the bandwidth) was chosen automatically using the mean square error optimal method that balances potential bias from wider bandwidth against increased variance of a smaller bandwidth [42].

To test the robustness of the predicted effect estimates to using different population sizes to create the model, we varied the bandwidth from 40 to 120 points to either side of the score cut-point, giving population sizes from 1980 to 4489. The minimum of 40 was used to ensure inclusion of data points after the second score cut-point of 20. Besides varying the bandwidth, all other parameters were identical to those in the primary model results shown above.

The third columns in Figs. 3 and 4 show the predicted effect sizes of attending a selective schooling for the range of populations to either side of the cut-point (81 effect size predictions are shown per outcome). The predicted effects are consistent across the range and gain precision as the population size increases. These results support a small negative effect of selective secondary schooling on self-reported outcomes across the range of bandwidths (Fig. 3c, f, i), but there is no clear evidence for an effect on hospital or mortality outcomes (Fig. 4c, f, i). For all outcomes (except the largest bandwidths for general health in mid-life), the confidence intervals of the estimates cross zero effect.

**Fig. 5:**
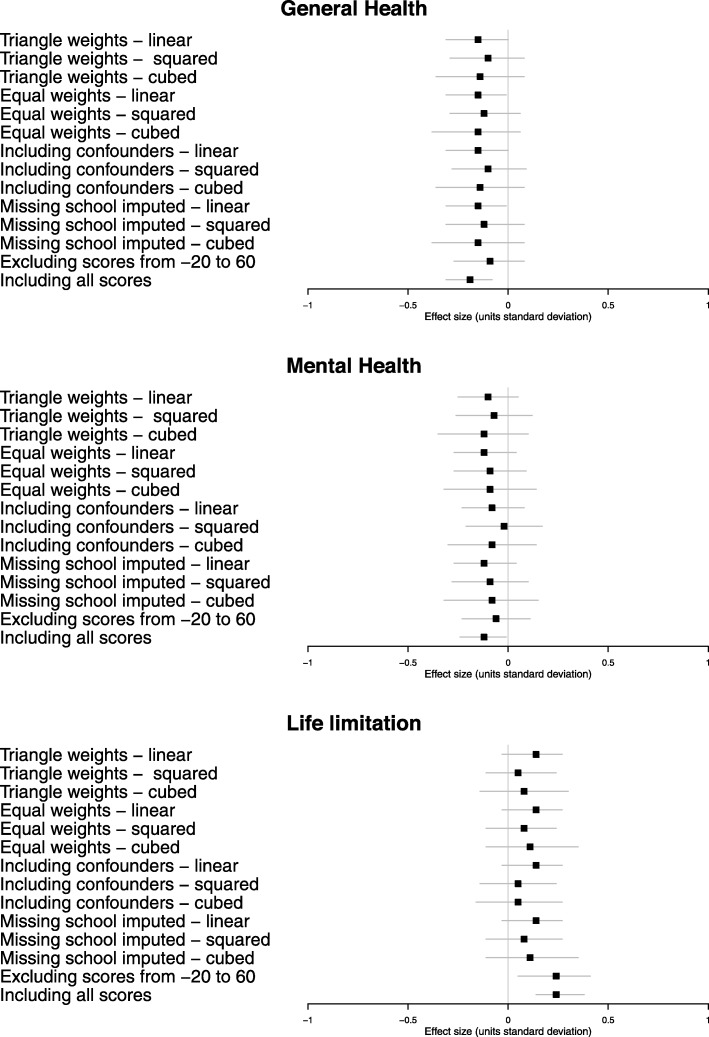
Sensitivity analysis of the estimated effect of selective schooling on the health outcomes self-reported at mid-life. Analyses are (1) using higher weights for those closer to the cut-point (triangular); (2) using equal weights; (3) including confounders of sex, IQ at age 9, and father’s occupation; (4) including the 3048 people in the base sample for whom secondary school was not known using inverse-probability weighting; (5) excluding those whose probability of attending selective secondary was not 0 or 100% (donut analysis); and (6) including the entire population equally weighted. Each of these variations was evaluated for first-, second-, or third-order polynomials of the 11+ exam score. Each sensitivity analysis effect estimate is represented as a point with associated 95% confidence intervals

**Fig. 6:**
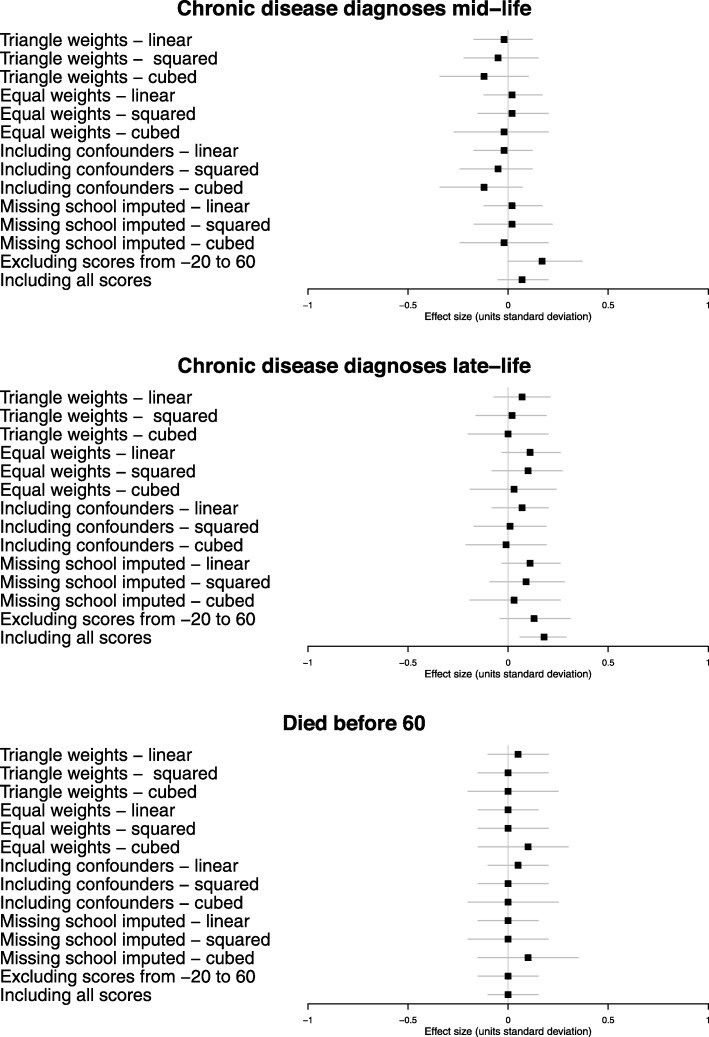
Sensitivity analysis of the estimated effect of selective schooling on the health outcomes from hospital admission and death records. Analyses are (1) using higher weights for those closer to the cut-point (triangular); (2) using equal weights; (3) including confounders of sex, IQ at age 9, and father’s occupation; (4) including the 3048 people in the base sample for whom secondary school was not known using inverse-probability weighting; (5) excluding those whose probability of attending selective secondary was not 0 or 100% (donut analysis); and (6) including the entire population equally weighted. Each of these variations was evaluated for first-, second-, or third-order polynomials of the 11+ exam score. Each sensitivity analysis effect estimate is represented as a point with associated 95% confidence intervals

Figures 5 and 6 show the estimated effect of attending selective secondary school on the health outcomes: (1) using higher weights for those closer to the cut-point (triangular); (2) using equal weights; (3) including confounders of sex, IQ at age 9, and father’s occupation; (4) including the 3048 people in the base sample for whom secondary school was not known using inverse-probability weighting; (5) excluding those whose probability of attending selective secondary was not 0 or 100% (donut analysis); and (6) including the entire population equally weighted. Each of these variations was evaluated for first-, second-, or third-order polynomials of the 11+ exam score. Effect sizes, confidence intervals and *p* values for these estimates are given in Additional Table 2.

These sensitivity analyses consistently produced estimates of a small (but imprecise) negative effect of selective schooling on the self-reported health outcomes; no effect on the likelihood of dying prematurely or on chronic disease diagnoses at mid-life and negative but imprecise estimates for chronic disease diagnoses in later life—all consistent with the primary model.

## Discussion

We found that people who attended selective secondary school had better health. This was true of health measures that were self-reported or derived from government records, whether measured in mid-life or later life. Importantly, we also found that in addition to better health, people who attended selective secondary school had more advantaged socioeconomic backgrounds, higher childhood IQs, and higher likelihood of getting a university degree.

However, we did not find evidence that selective schooling *itself* improved health. We measured selective schooling’s effect on self-reported health in mid-life, chronic disease burden in mid-life and late-life derived from hospital records, and the likelihood of dying prematurely. Estimates of selective schooling’s effect on these measures were primarily none or negative. This lack of a positive influence of selective secondary schooling on health was consistent despite varying a wide range of the model’s assumptions. The effect sizes ranged from 0.10 to 0.15 standard deviations worse for self-reported health, and from 0.02 standard deviations better to 0.07 worse for record-derived health. None of these results reach the 0.25 standard deviation difference marking the threshold of an educational intervention of substantive importance [47]. Furthermore, the estimates were too imprecise to allow us to conclude selective schooling was detrimental without further study.

We were able to isolate the effect of selective schooling by using a natural experiment in secondary school assignment in the UK. The Aberdeen Children of the 1950s study began during the era of school assignment by test score and collected data on an entire large population, removing the bias that can occur in voluntary enrolment studies. The population was assigned to school using the same test and criteria, removing measurement error present when considering school assignment in country-wide studies where test and threshold scores often vary. Because the study has run for the past 50 years, we were able to assess the effect of schooling on health long term, and Scotland’s data sharing policies mean we were able to link to routinely collected data rather than rely solely on self-reports, removing participation bias and increasing the range of health outcome measures. We found no evidence that associations between confounders and cognitive ability changed at the assignment point, suggesting risk of confounding bias is low in the design. There was also no evidence that cognitive ability’s association with questionnaire response or being traced in the NHS changed at the assignment point, suggesting risk of participation bias is also low. Furthermore, sensitivity analyses controlling for confounders and weighted for non-response to the survey showed little impact on the results. This evidence together suggests the natural experiment was well-identified and the design well-controlled for confounding.

In evaluating these results within the body of work on education and health, we see that previous research (including in this cohort) has shown that attending selective secondary school increases length of time in school [23]. However, there is mixed evidence that this has a causal effect on health. One natural experiment study exploiting the legislated rising of the school leaving age in Britain in 1972 suggests it led to reduced mortality risk and better health outcomes in UK Biobank participants [13]. In contrast, and in line with this work, a study using the same natural experiment and a similar one in 1947 found little effect on mortality across England and Wales [11].

If higher income is a mechanism by which health advantage is gained, this pathway may not have existed in our population. Previous research did not find an effect of selective schooling on income in this cohort [23], possibly due to the economic boom related to North Sea oil production as they entered employment, when qualifications were less important for labour market success. However, a recent study using the 1958 English birth cohort uses a proxy of the 11+ score in a regression discontinuity design and found an effect of selective secondary schooling on educational attainment, but no effect on income or health, in line with our findings [48].

Despite the strong natural experiment design, our results may still be biased. The study was designed to calculate the effects specific to people with an 11+ score at the assignment cut-point, so any heterogeneous impacts of selective schooling will be missed. Furthermore, despite having a large sample, the design favours reduction of confounding bias over precision, and small effects may have been missed. We sought to mitigate these limitations by looking at multiple health outcomes and using comprehensive sensitivity analyses, which showed consistent direction of effect.

## Conclusions

We did not find evidence that selective schooling had a positive effect on self-reported or record-derived health in mid-life or later life in a large Scottish population. The rationale given for expanding selective secondary schooling in the UK is centred on educational advantages, but findings from this and similar work suggest that the advantages should not be extended to include health benefits. As population well-being is often judged by economic and health success, these findings suggest that the separating school-children by ability is not an efficient way to make changes.

## Supplementary information

**Additional file 1: Table S1.** Study variables. (Excel spreadsheet) giving the source, description, time frame, and possible values of all the variables received from the Aberdeen Children of the 1950s study or from NHS hospital admissions records.

**Additional file 2:** Study code. (Plain text file) giving the metadata and all code used for analysis and generation of figures and tables in this paper.

**Additional file 3: Figure S1.** Assessment of the smoothness of the confounding variables across the selective schooling cut-point. Population is the study sample (*n* = 5039) and points are means of 50 people. Figures A, D and G in first column show the 11+ exam score vs the confounder. Figures B, E and H in the second column show the same data with the population separated by selective (open orange triangles) and non-selective (open blue circles) secondary school attendance. The line shows the estimated effect size of secondary school attendance at the cut-point extrapolated across the optimal bandwidth. Figures C, F and I in the third column show re-estimates of the effect size of secondary school attendance for a range of population sizes around the cut-point (bandwidth sizes). The effect size estimate at the predicted optimal bandwidth (reported in Table 3) is shown as a dashed line.

**Additional file 4: Table S2.** Effect size estimates from sensitivity analyses. (Excel spreadsheet) giving estimated effect of attending selective secondary school on the health outcomes: 1) using higher weights for those closer to the cut-point (triangular); 2) using equal weights; 3) including confounders of sex, IQ at age 9 and father’s occupation; 4) including the 3048 people in the base sample for whom secondary school was not known using inverse-probability weighting; 5) excluding those whose probability of attending selective secondary was not 0 or 100% (donut analysis); and 6) including the entire population equally weighted. Includes evaluation for first, second, or third order polynomials of the 11+ exam score. Also includes effect sizes, confidence intervals, and *p*-values calculated in units of standard deviation of the the study sample.
