# Supplementary figures and images for: The long-term health effects of attending a selective school: a natural experiment
Source: BMC Med. 2020 Apr 3;18:77. doi: 10.1186/s12916-020-01536-7 (PMC7118818; doi:10.1186/s12916-020-01536-7)

A

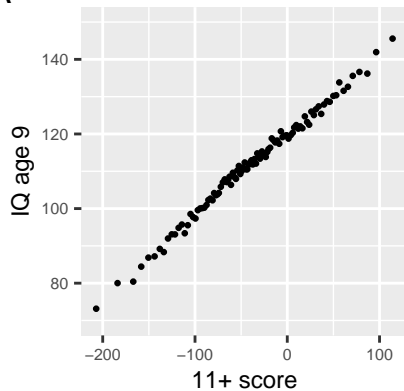

B

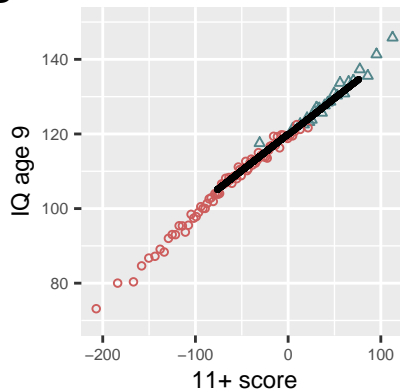

C

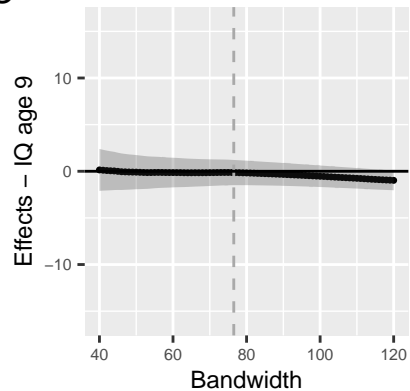

D

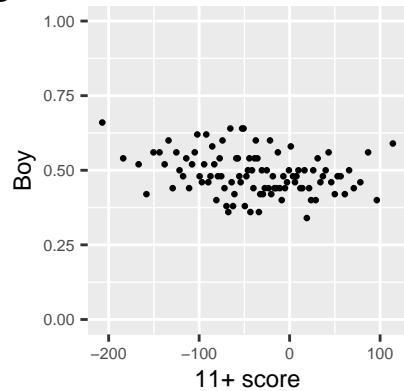

E

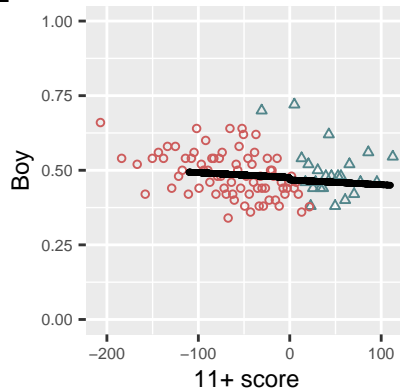

F

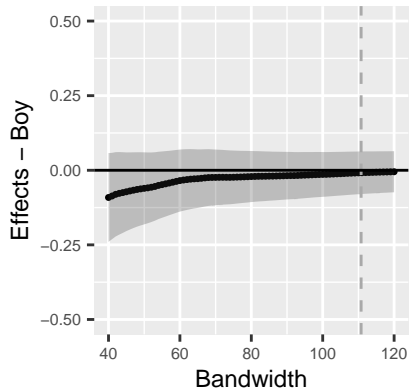

G

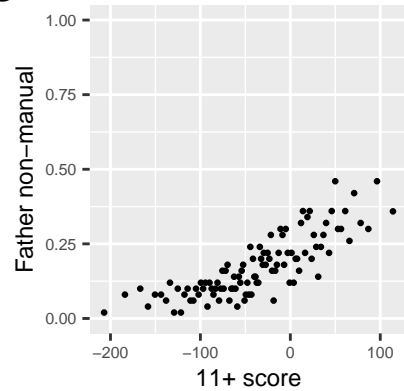

H

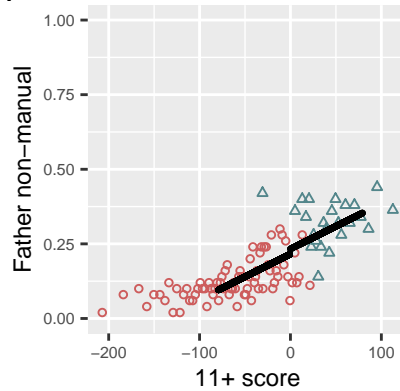

I

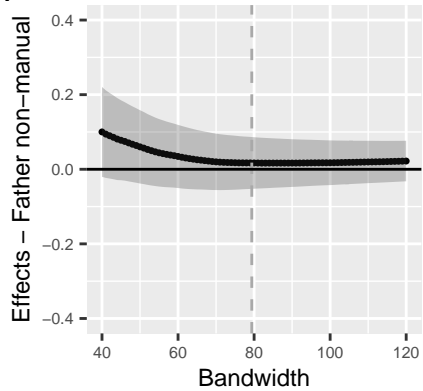

Supplement: Supplementary file 3 — Additional file 3: Figure S1. Assessment of the smoothness of the confounding variables across the selective schooling cut-point. Population is the study sample (n = 5039) and points are means of 50 people. Figures A, D and G in first column show the 11+ exam score vs the confounder. Figures B, E and H in the second column show the same data with the population separated by selective (open orange triangles) and non-selective (open blue circles) secondary school attendance. The line shows the estimated effect size of secondary school attendance at the cut-point extrapolated across the optimal bandwidth. Figures C, F and I in the third column show re-estimates of the effect size of secondary school attendance for a range of population sizes around the cut-point (bandwidth sizes). The effect size estimate at the predicted optimal bandwidth (reported in Table 3) is shown as a dashed line. [file 12916_2020_1536_MOESM3_ESM.pdf]
